# Author Correction: Atomic layer deposited Pt-Ru dual-metal dimers and identifying their active sites for hydrogen evolution reaction

**DOI:** 10.1038/s41467-019-13466-x

**Published:** 2019-11-26

**Authors:** Lei Zhang, Rutong Si, Hanshuo Liu, Ning Chen, Qi Wang, Keegan Adair, Zhiqiang Wang, Jiatang Chen, Zhongxin Song, Junjie Li, Mohammad Norouzi Banis, Ruying Li, Tsun-Kong Sham, Meng Gu, Li-Min Liu, Gianluigi A. Botton, Xueliang Sun

**Affiliations:** 1 Department of Mechanical and Materials Engineering, The University of Western Ontario, London, ON N6A 5B9 Canada; 2 Beijing Computational Science Research Center, Beijing, 100193 China; 3 School of Physics, Beihang University, Beijing, 100083 China; 4 Department of Materials Science and Engineering, McMaster University, Hamilton, ON L8S 4L8 Canada; 5 Canadian Light Source Inc, Saskatoon, SK S7N 2V3 Canada; 6 Department of Materials Science and Engineering, Southern University of Science and Technology, Shenzhen, 518055 China; 7 Department of Chemistry, University of Western Ontario, London, ON N6A 5B7 Canada

**Keywords:** Catalyst synthesis, Hydrogen fuel, Electrocatalysis

Correction to: *Nature Communications* 10.1038/s41467-019-12887-y, published online 30 October 2019.

The original version of this Article contained an error in the author affiliations.

Li-Min Liu was incorrectly associated with ‘Beijing Computational Science Research Center, Beijing 100193, China.’

This has now been corrected in both the PDF and HTML versions of the Article.

