# Supplementary material for: Atomic layer deposited Pt-Ru dual-metal dimers and identifying their active sites for hydrogen evolution reaction
Source: Nat Commun. 2019 Oct 30;10:4936. doi: 10.1038/s41467-019-12887-y (PMC6821730; doi:10.1038/s41467-019-12887-y)
Supplement: Supplementary file 1 — Supplementary Information [file 41467_2019_12887_MOESM1_ESM.pdf]

Supplementary Information

Atomic Layer Deposited Pt-Ru Dual-Metal Dimers and Identifying Their Active Sites for Hydrogen Evolution Reaction

Lei Zhang,[1,[a]] Rutong Si,[1,[c][g]] Hanshuo Liu,[1,[b]] Ning Chen,[d] Qi Wang,[e] Keegan Adair,[a] Zhiqiang Wang,[f] Jiatang Chen,[f] Zhongxin Song,[a] Junjie Li,[a] Mohammad Norouzi Banis,[a] Ruying Li,[a] Tsun-Kong Sham,[f] Meng Gu,[e] Li-Min Liu,* [c][g] Gianluigi A. Botton,*[b] and Xueliang Sun*[a]

[a] Department of Mechanical and Materials Engineering, The University of Western Ontario, London, ON N6A 5B9, Canada

[b] Department of Materials Science and Engineering, McMaster University, Hamilton, ON L8S 4L8, Canada

[c] Beijing Computational Science Research Center, Beijing 100193, China

[d] Canadian Light Source Inc. Saskatoon, SK, S7N 2V3, Canada

[e] Department of Materials Science and Engineering, Southern University of Science and Technology, Shenzhen, 518055, China.

[f] Department of Chemistry, University of Western Ontario, London, ON, N6A 5B7, Canada

[g] School of Physics, Beihang University, Beijing, 100083, China

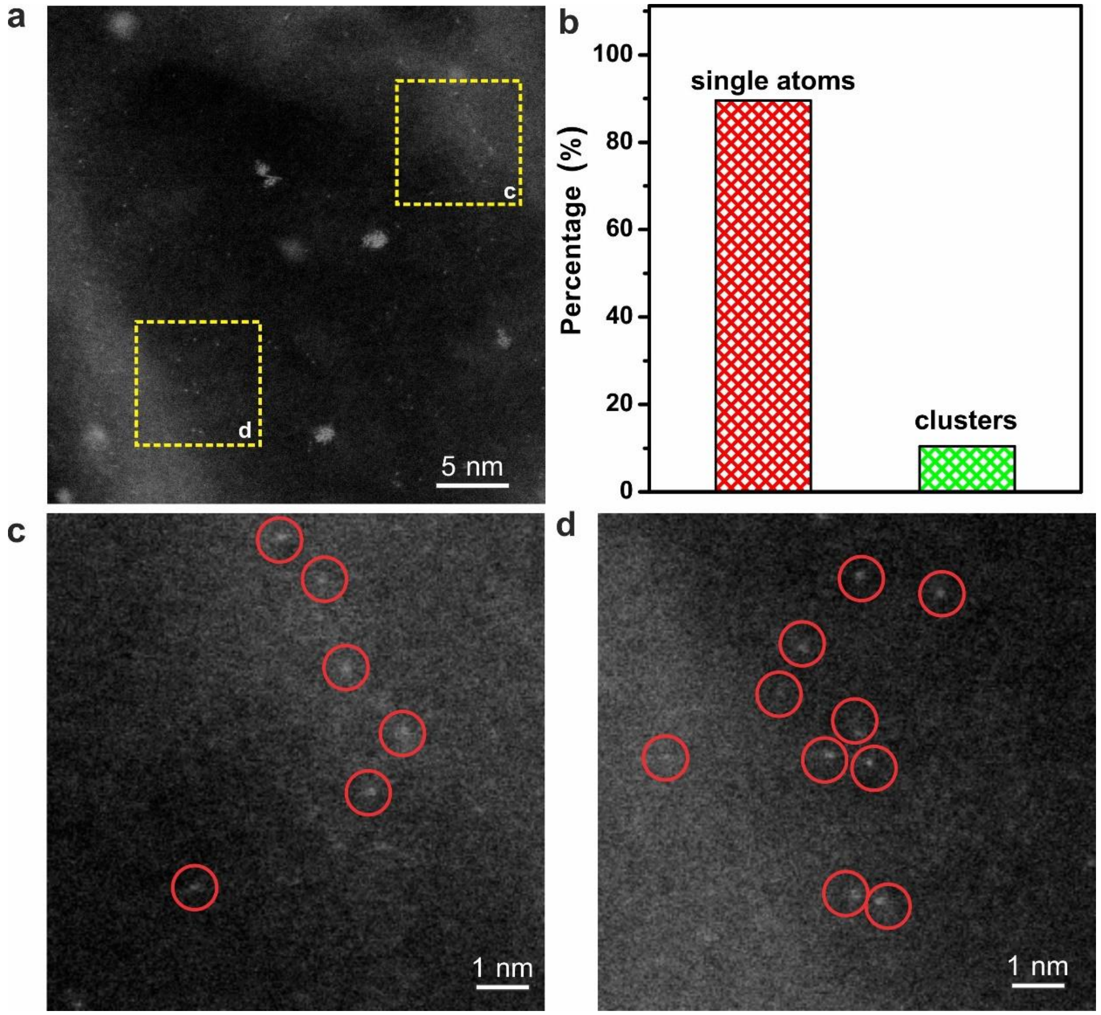

Supplementary Figure 1. Characterizations of Pt single atoms. (a) Aberration-corrected HAADF-STEM image of Pt single atoms. (b) Distribution histogram showing the ratio between single atoms and clusters. (c, d) Enlarged aberration-corrected HAADF-STEM images of Pt single atoms.

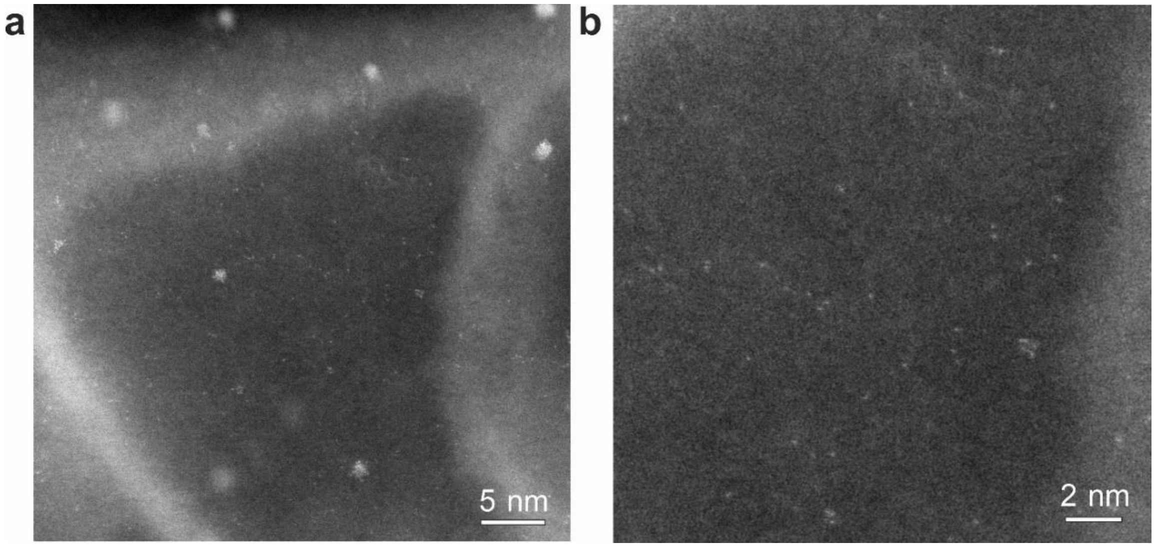

Supplementary Figure 2. Characterizations of Pt single atoms after heating at 270 ^{o}C in ALD chamber for 1 h. (a) Aberration-corrected HAADF-STEM image and (b) enlarged HAADF-STEM image of Pt single atoms after heating at 270 ^{o}C in ALD chamber for 1 h.

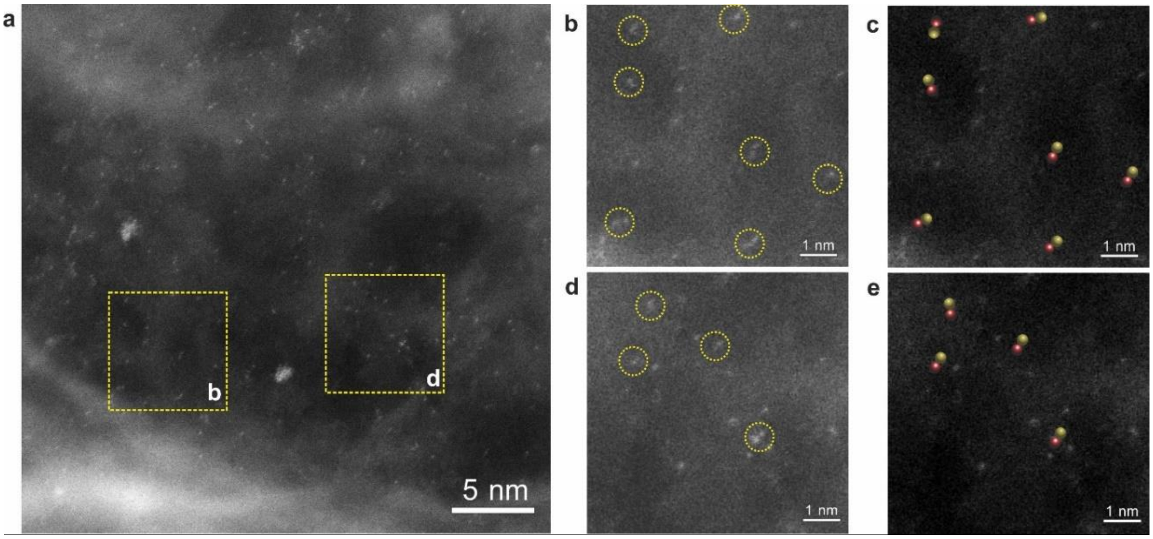

Supplementary Figure 3. Characterizations of Pt-Ru dimers. (a) Aberration-corrected HAADF-STEM images of Pt-Ru dimers/NCNTs. (b-e) Aberration-corrected HAADF-STEM images of Pt-Ru dimers/NCNTs located at different positions.

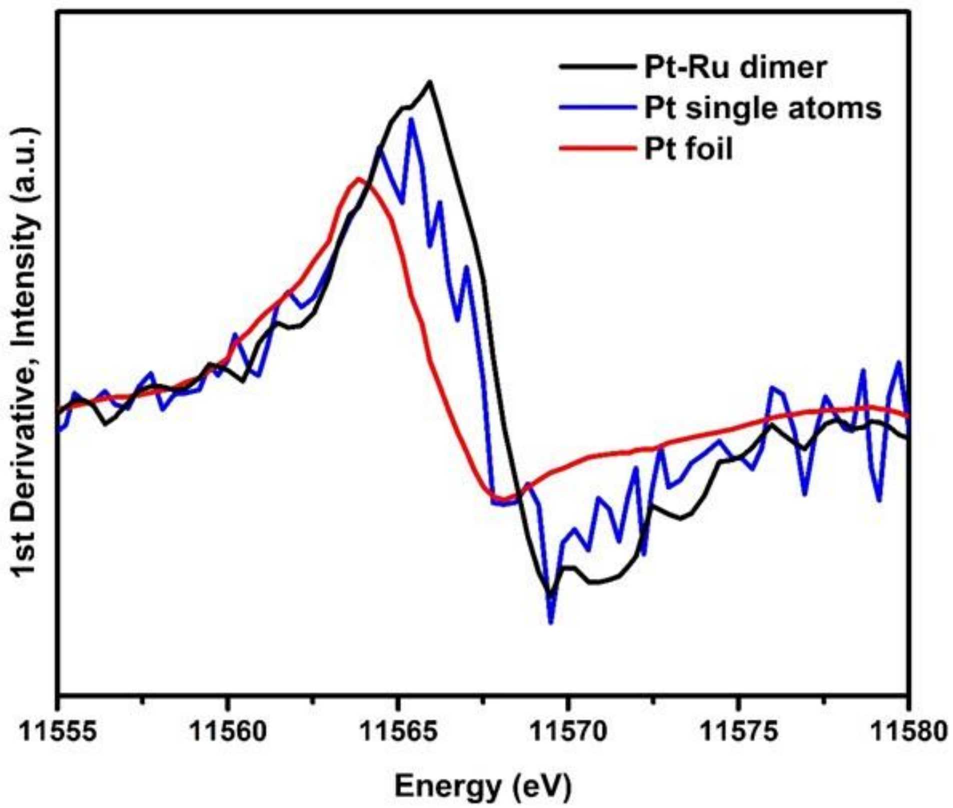

Supplementary Figure 4. The first derivative of the XANES spectrum at Pt L3 edge.

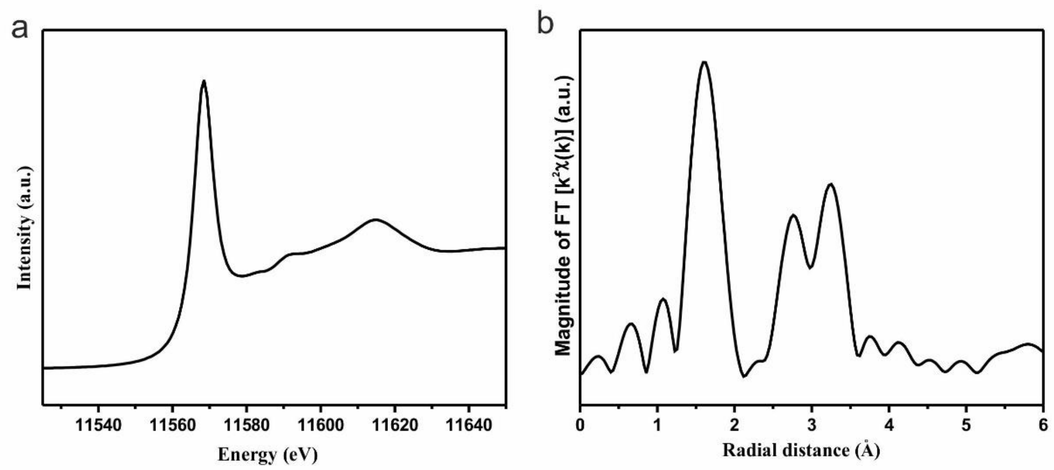

Supplementary Figure 5. X-ray absorption studies of the PtO_2. (a) The normalized XANES spectra at the Pt L3-edge of the PtO_2. (b) Corresponding K2-weighted magnitude of Fourier transform spectra from EXAFS of PtO_2.

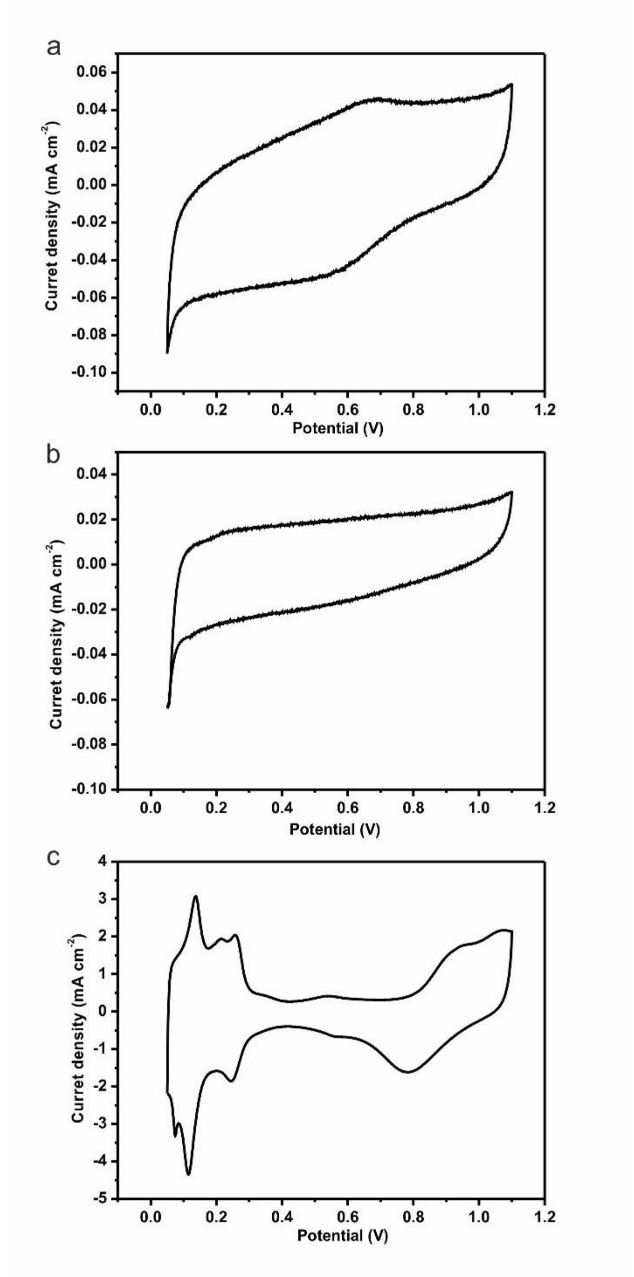

Supplementary Figure 6. Cyclic voltammograms (CV) of the catalysts. CV curves of (a) Pt-Ru dimers, (b) Pt single atoms and (c) Pt/C catalysts. The current densities were normalized to the geometric area of the RDE (0.196 cm^2).

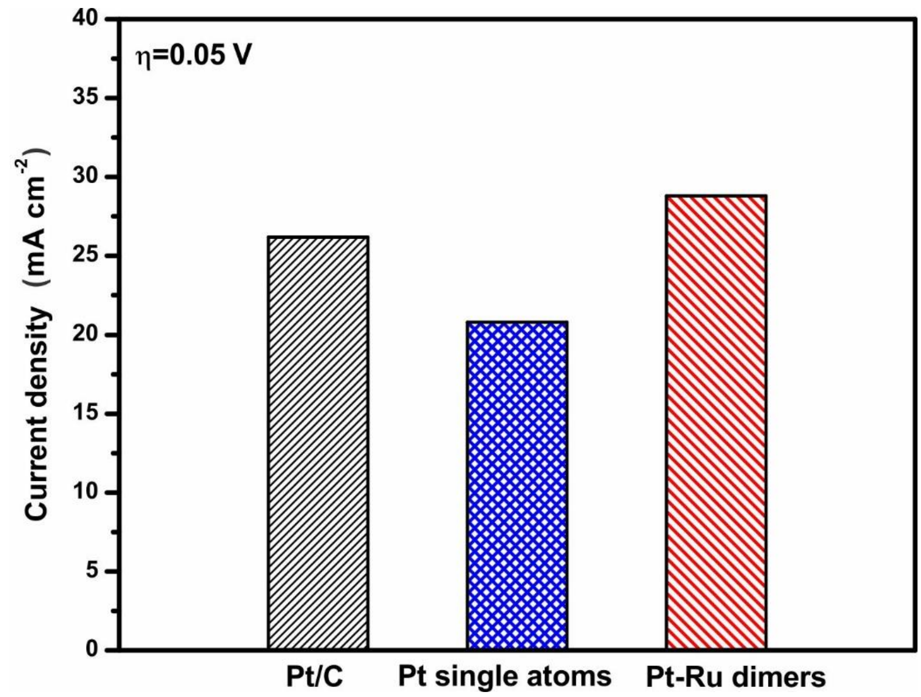

Supplementary Figure 7. The specific activities at 0.05 V of Pt-Ru dimers, Pt single atoms and Pt/C catalysts, which were normalized to the geometric area of the RDE (0.196 cm^2).

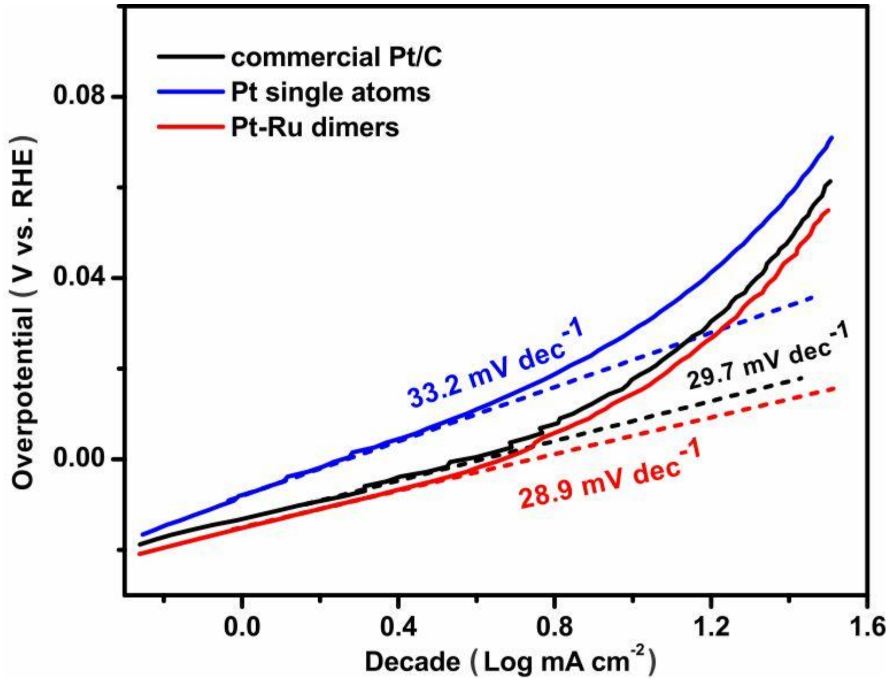

Supplementary Figure 8. Tafel plots recorded on Pt-Ru dimers, Pt single atoms and Pt/C catalysts, respectively.

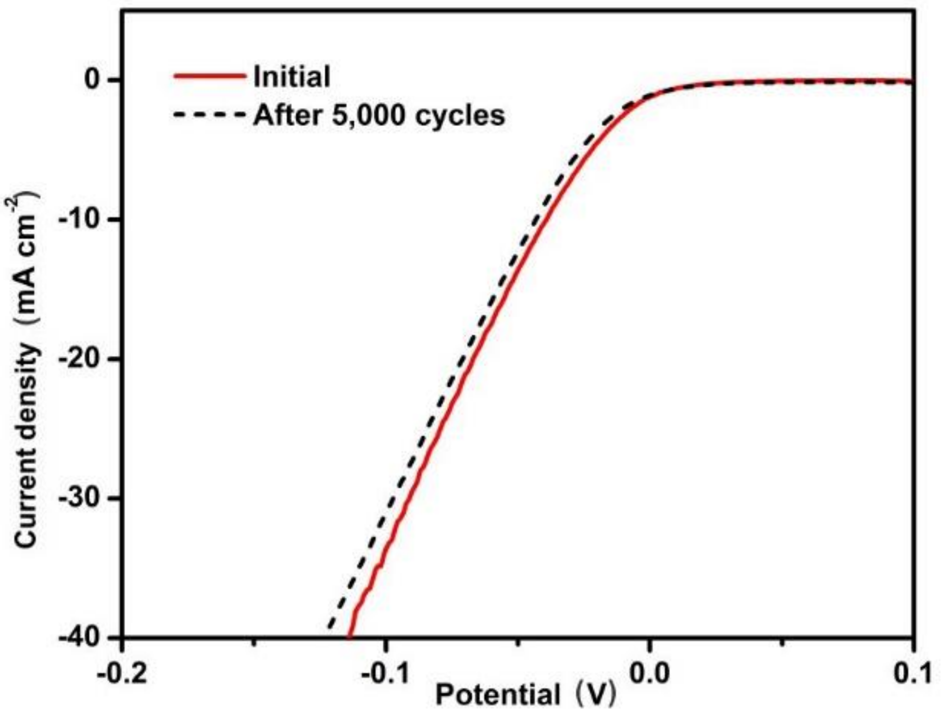

Supplementary Figure 9. Durability measurement of the Pt single atom catalysts.

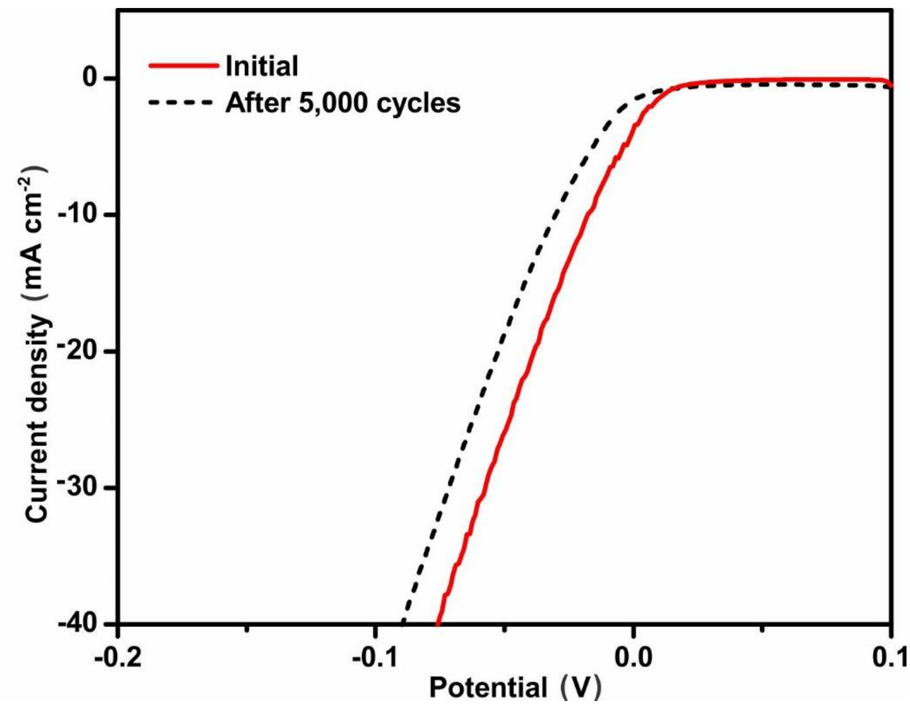

Supplementary Figure 10. Durability measurement of the Pt/C catalysts.

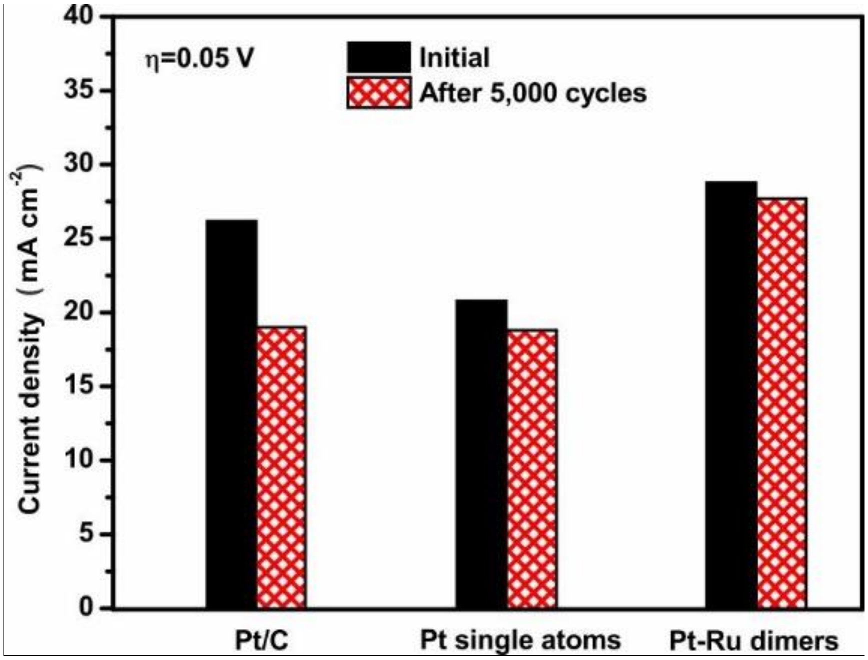

Supplementary Figure 11. Normalized specific activity at 0.05 V of Pt-Ru dimers, Pt single atoms and Pt/C catalysts before and after durability test.

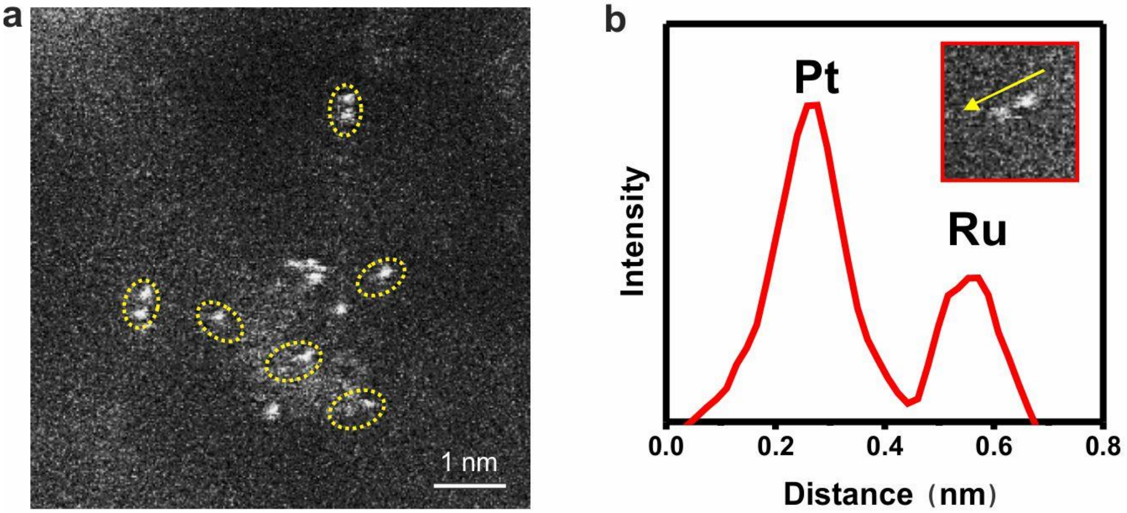

Supplementary Figure 12. Characterizations of Pt-Ru dimers after stability test. (a) Aberration-corrected HAADF-STEM images of Pt-Ru dimers/NCNTs after stability measurement. (b) The intensity profile obtained on one post-testing Pt-Ru dimer.

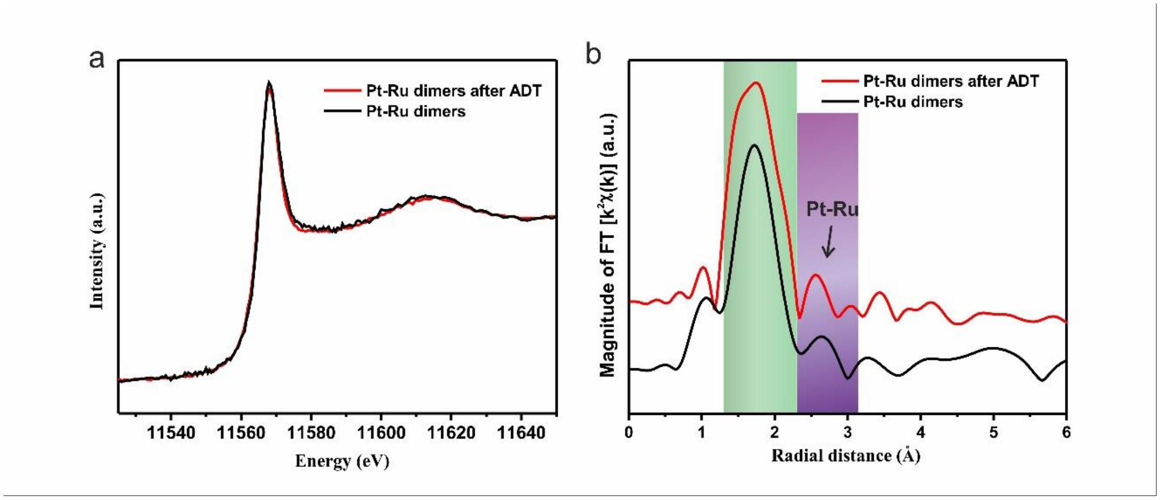

Supplementary Figure 13. X-ray absorption studies of Pt-Ru dimers after stability test. (a) The normalized XANES spectra at the Pt L3-edge of the Pt-Ru dimers before and after ADT tests. (b) corresponding K2-weighted magnitude of Fourier transform spectra from EXAFS of the Pt-Ru dimers before and after ADT tests.

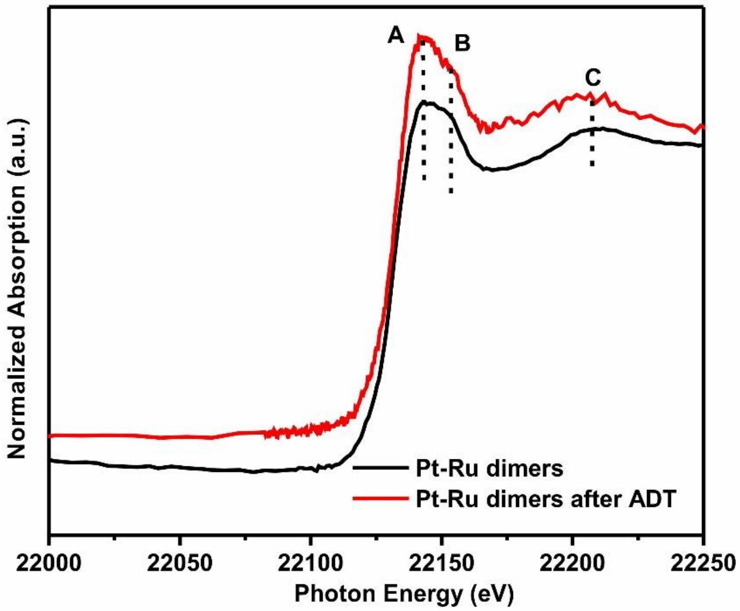

Supplementary Figure 14. X-ray absorption studies of Pt-Ru dimers after stability test. The normalized XANES spectra at the Ru K-edge of the Pt-Ru dimers before and after ADT tests.

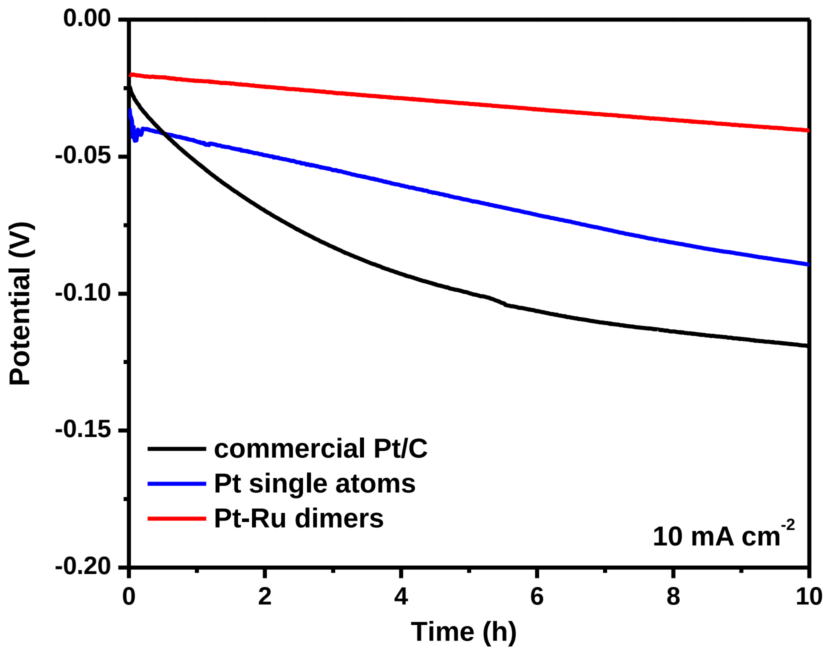

Supplementary Figure 15. Long-term stability tests. Stability of the Pt-Ru dimers, Pt single atoms and Pt/C catalysts for HER at 10 mA cm^{-2} V for 10 h.

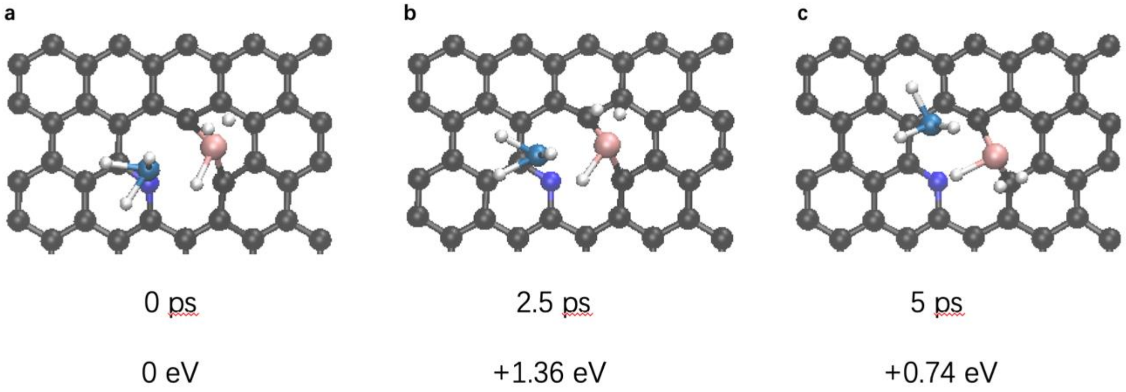

Supplementary Figure 16. The first principles molecular dynamics (FPMD) for understanding the stability of Pt-Ru dimers. (a) The FPMD results at 0ps of Pt-Ru dimer. (b) The results at 2.5ps of Pt-Ru dimer. (c) The results at 5ps of Pt-Ru dimer.

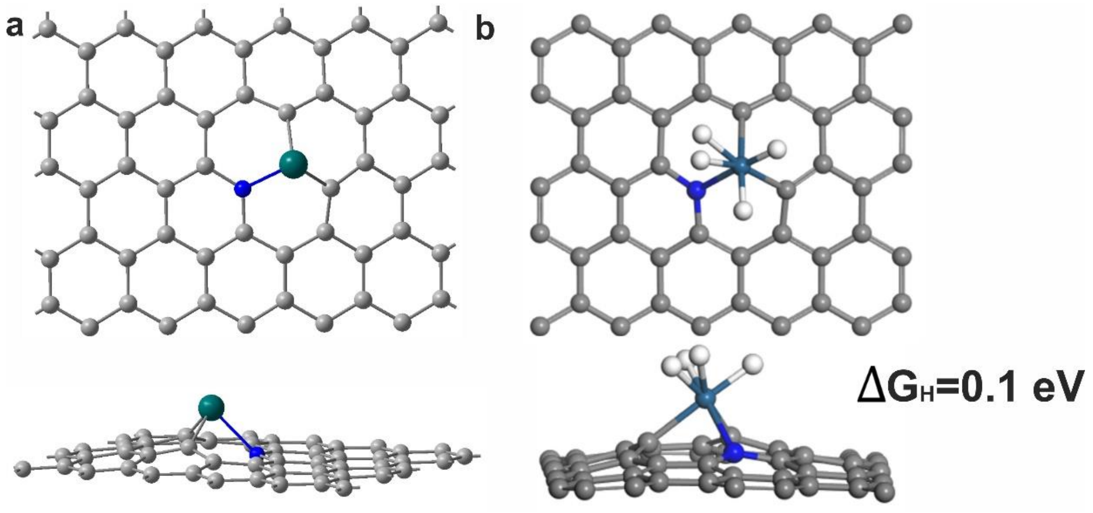

Supplementary Figure 17. The atomic model of Pt single atom structure. (a) The non-hydrogen adsorbed Pt single atom structure and (b) the structure with 4H adsorbed on Pt atom.

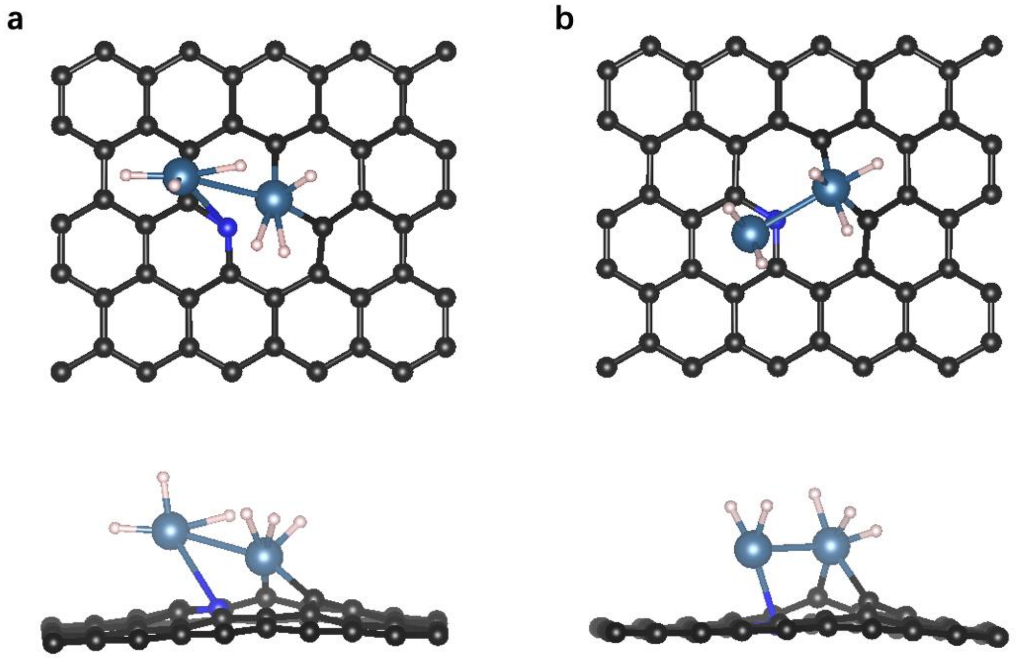

Supplementary Figure 18. The atomic model of Pt-Pt dimer structure. (a) Pt-Pt adsorbed with six H atoms; (b) Pt-Pt dimer adsorbed with five H atoms.

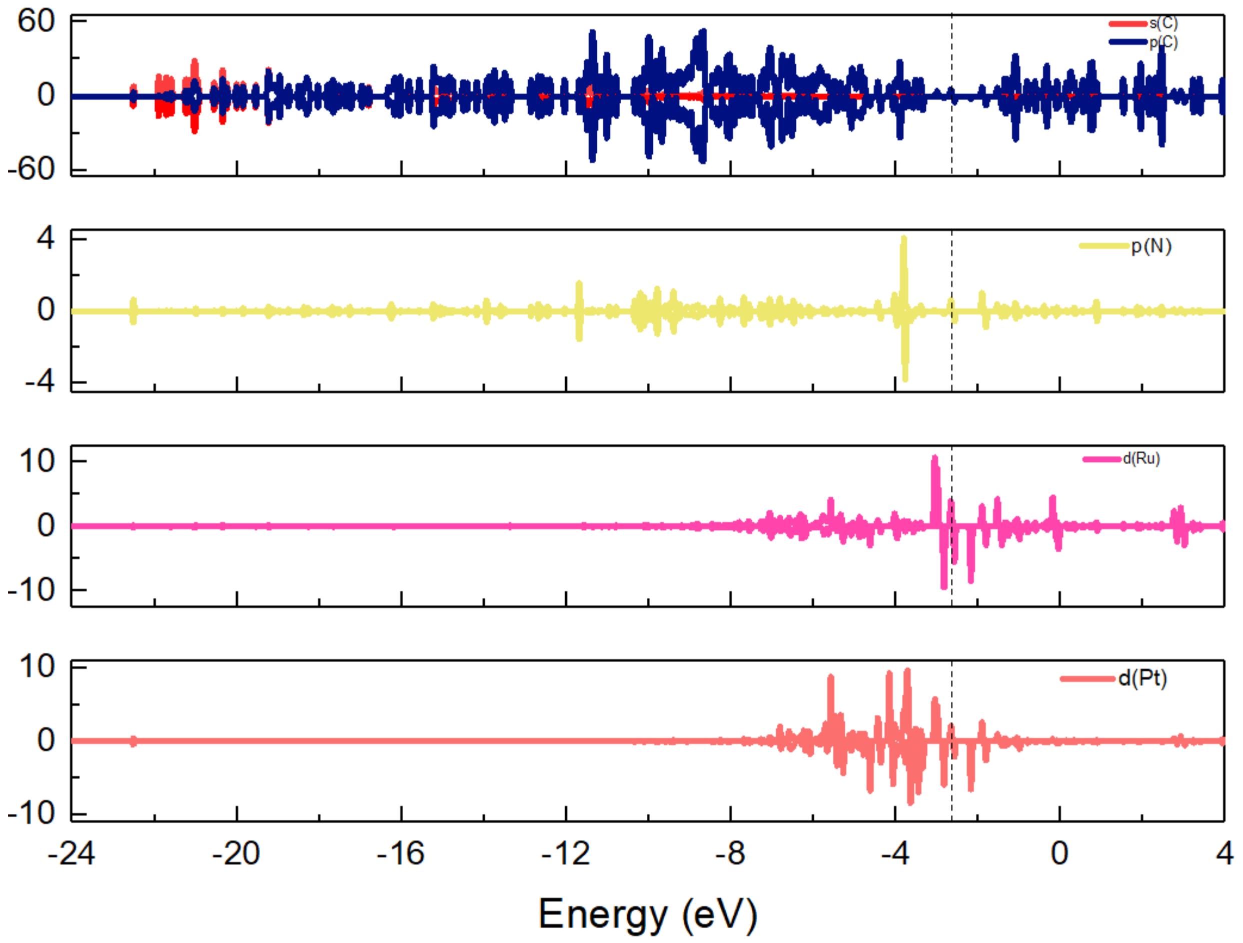

Supplementary Figure 19. The local density of states (LDOS) of C, N, Ru and Pt atoms in Pt(0H)Ru(0H) .

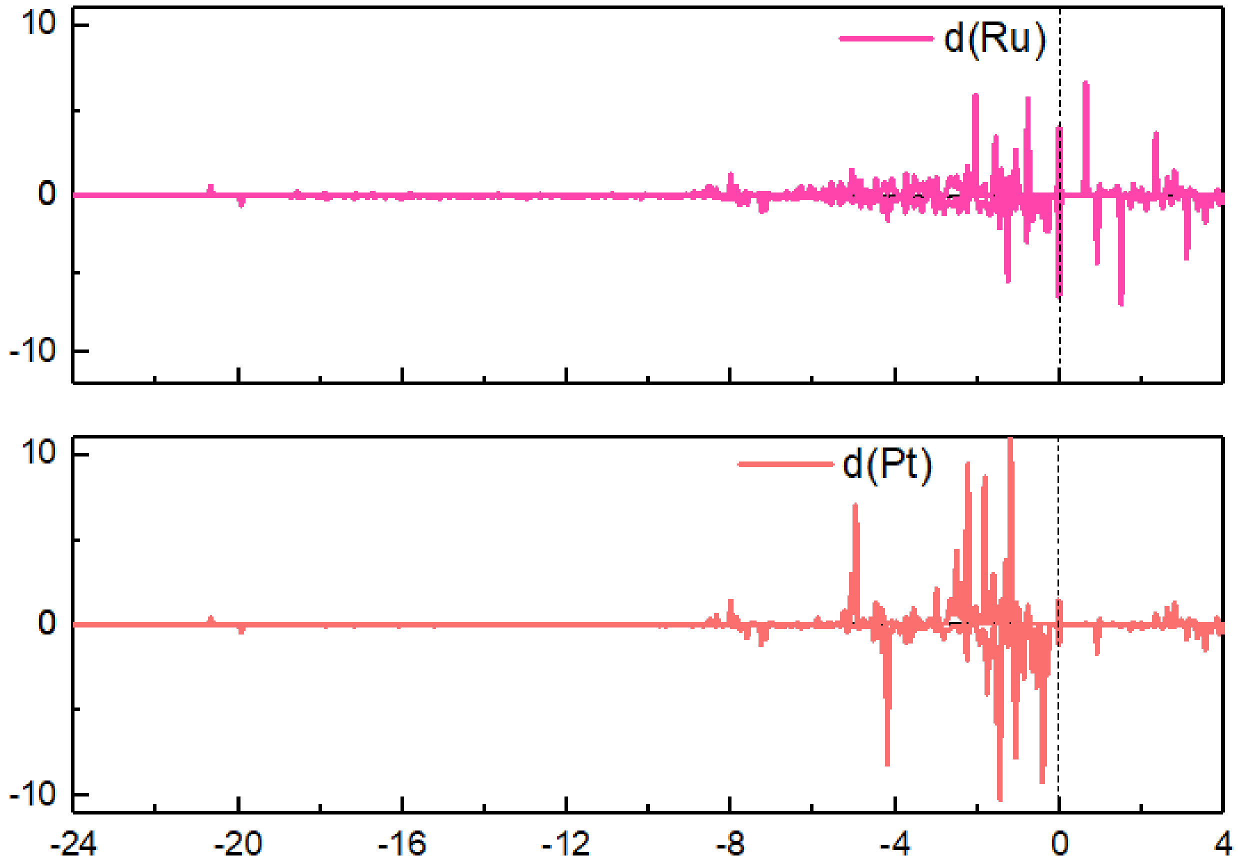

Supplementary Figure 20. The local density of states (LDOS) of Ru and Pt atoms in Pt(2H)Ru(2H) .

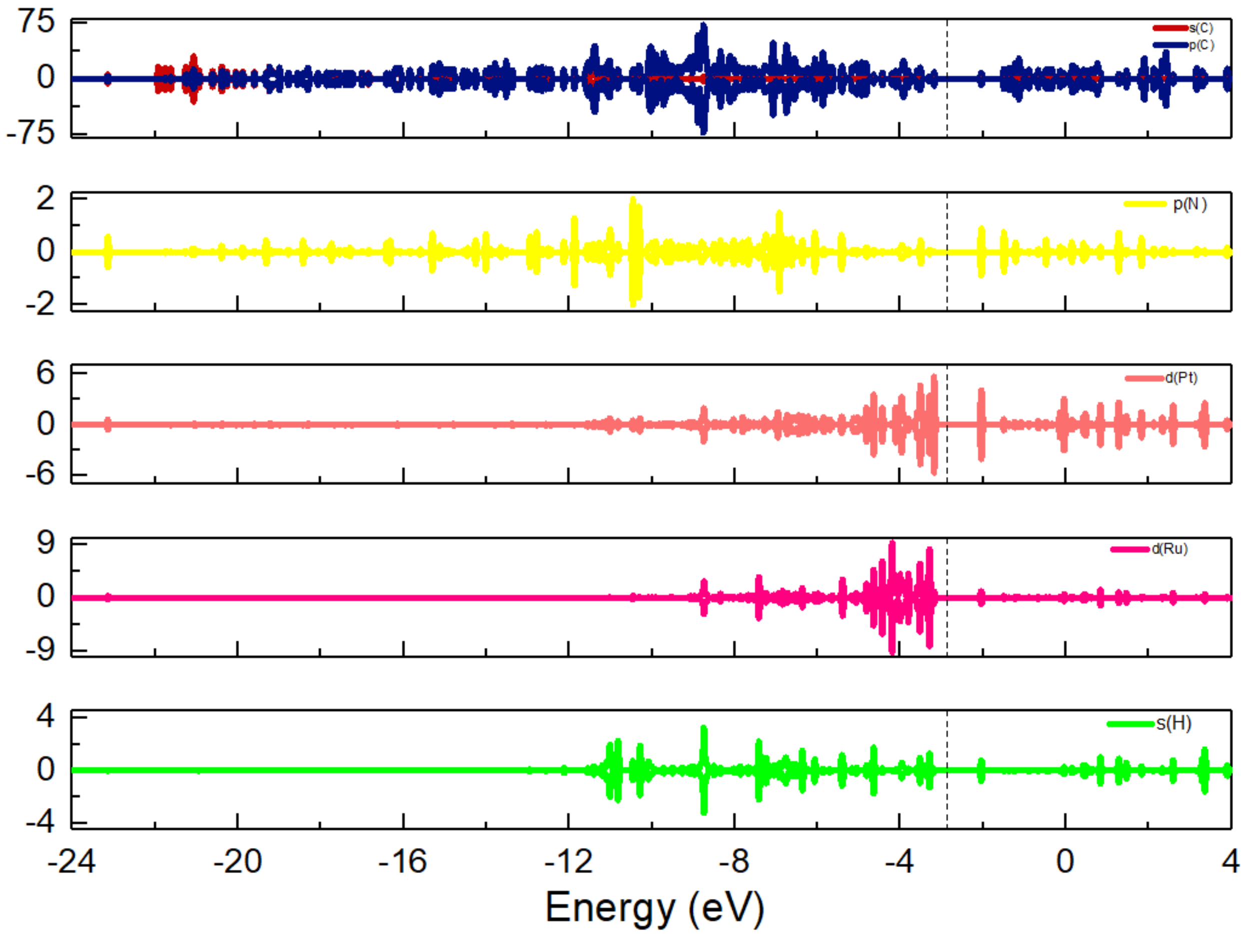

Supplementary Figure 21. The local density of states (LDOS) of H, C, N, Ru and Pt atoms in Pt(2H)Ru(3H).

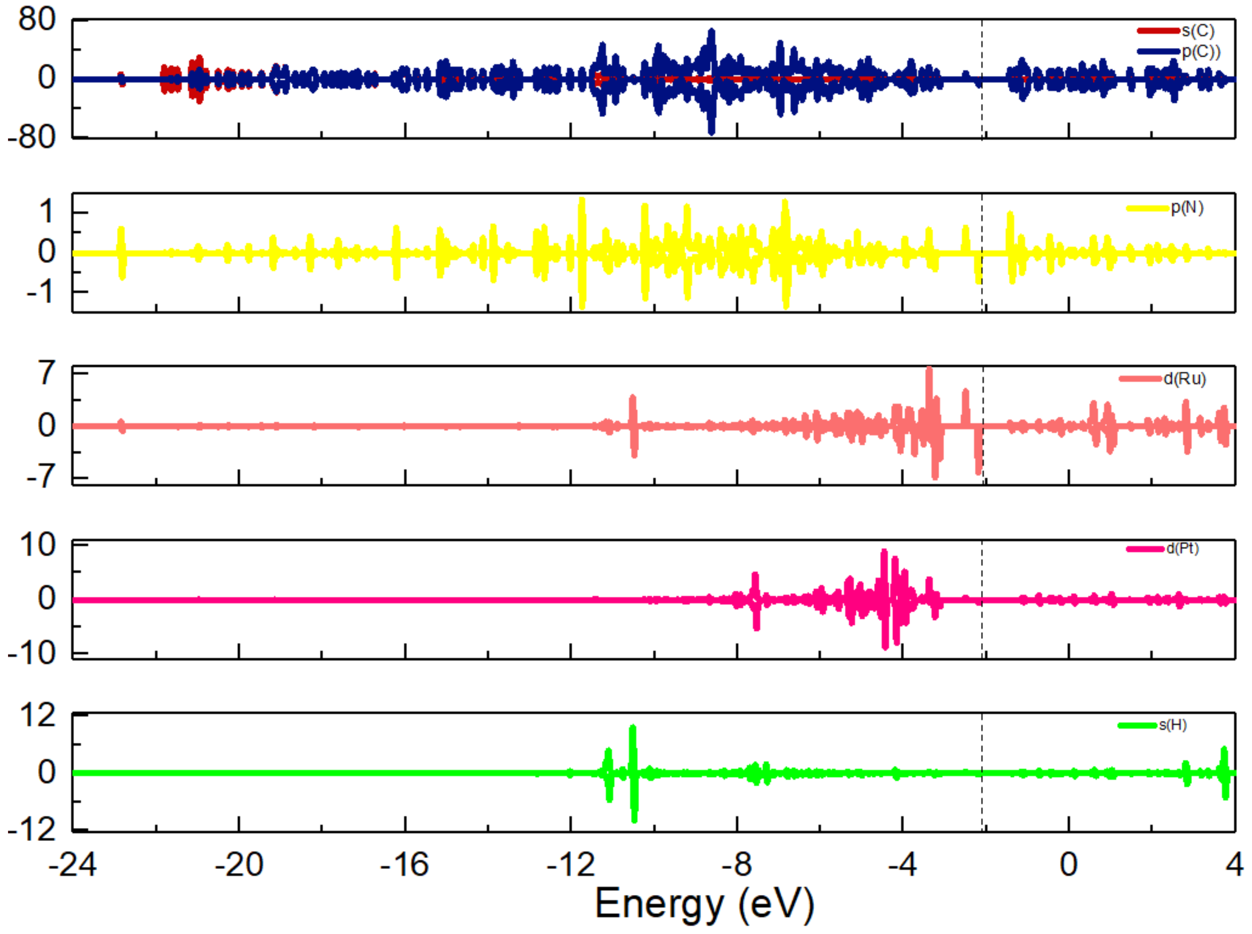

Supplementary Figure 22. The local density of states (LDOS) of H, C, N, Ru and Pt atoms in Pt(3H)Ru(3H).

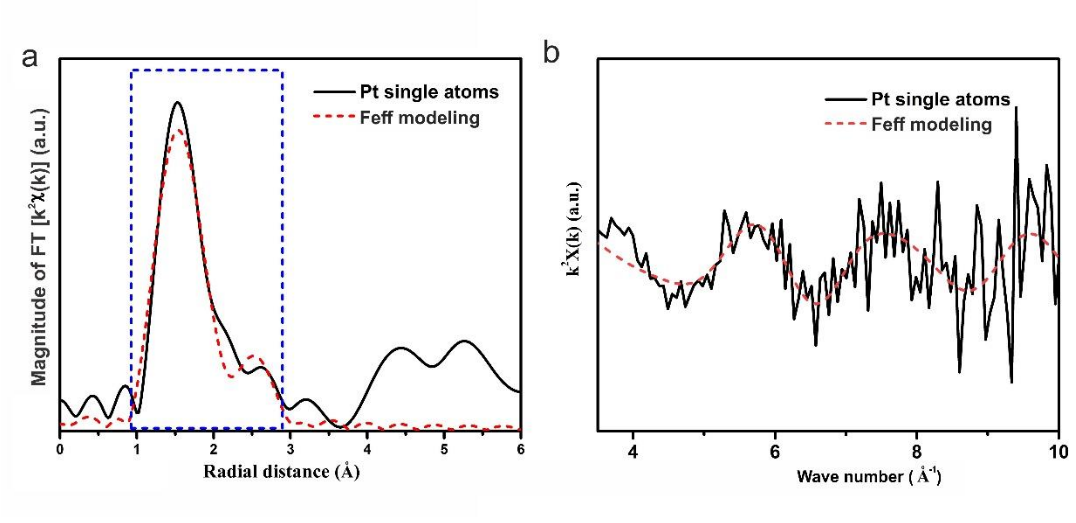

Supplementary Figure 23. R space and K space curve fitting on Pt single atoms. Comparison between experimental data from Pt single atoms and the Feff modeling based on the R space and K space curve fitting.

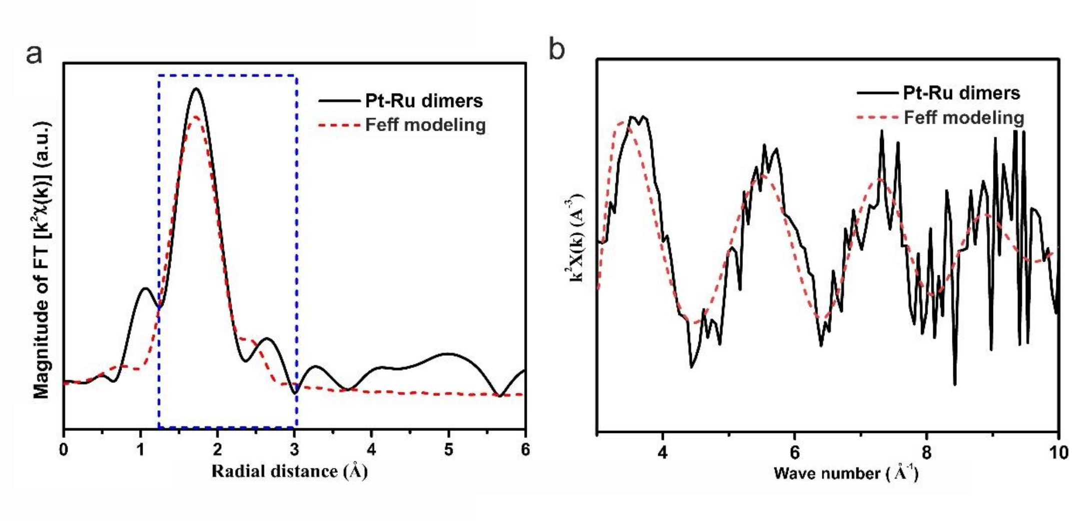

Supplementary Figure 24. Pt R space and K space curve fitting on Pt-Ru dimers. (a) Comparison in the magnitude of FT between Pt L_3 edge experimental data from Pt-Ru dimer and the Feff R space curve fitting; (b) comparison between experimental data and Feff modeling based on the R space result.

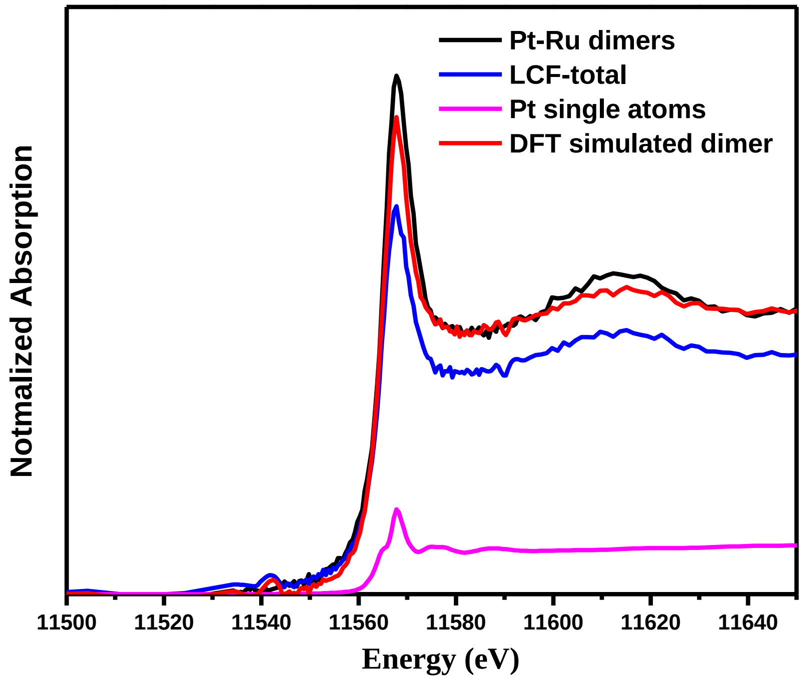

Supplementary Figure 25. The XANES linear combination fitting (LCF) results. The LCF analysis for Pt-Ru dimer XANES spectra by using DFT theoretical modeled XANES as one of standard spectrum, suggesting the existence of Pt-Ru dimer in the sample.

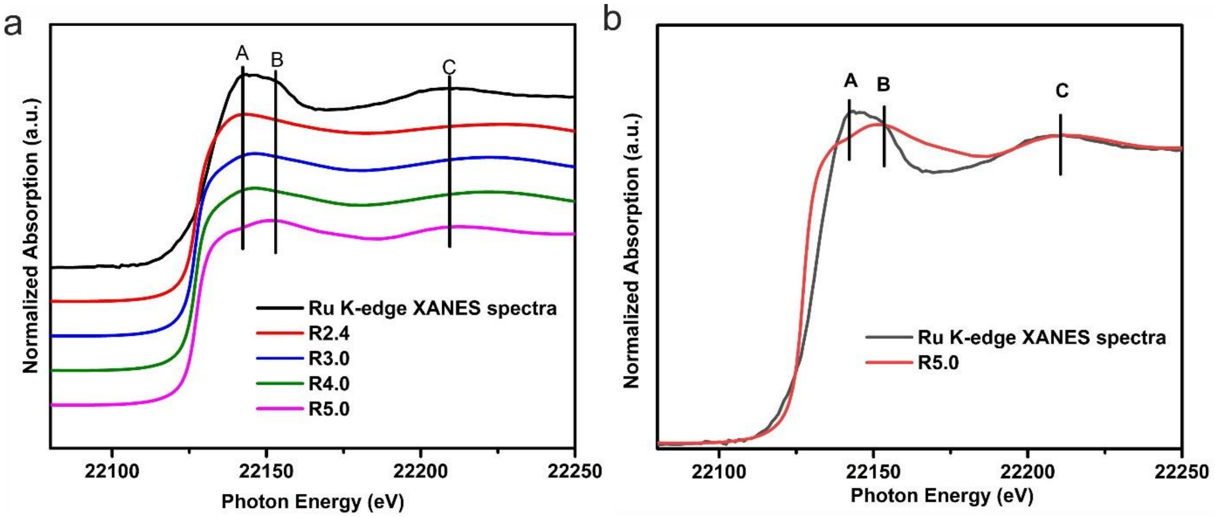

Supplementary Figure 26. The XANES spectra at the Ru K-edge and corresponding modeled theoretical XANES system with the Ru centered cluster system. (a) The cluster radius progressively increased from 2.4, 3.0, 4.0, to 5.0 Å, respectively. (b) The comparison between experimental XANES spectrum and the best-fit of Ru XANES modeling (Ru centered clusters with the radius of 5.0 Å).

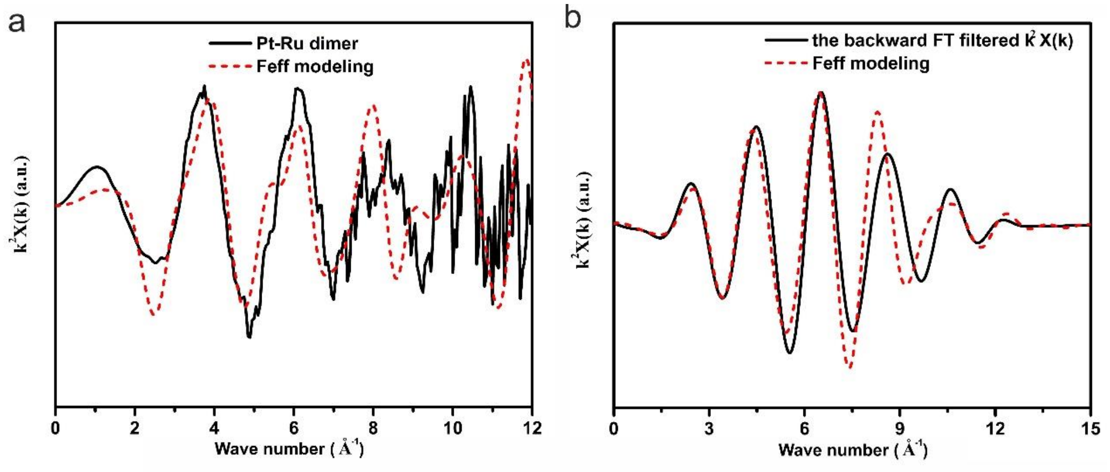

Supplementary Figure 27. Feff modeling based on the Ru centered cluster R5.0Å. Comparison in (a) raw data and (b) backward FT filtered $k^2\chi(k)$ between experimental data (black trace) and Feff modeling (red dash trace) based on the Ru centered cluster R5.0Å predicted by DFT model.

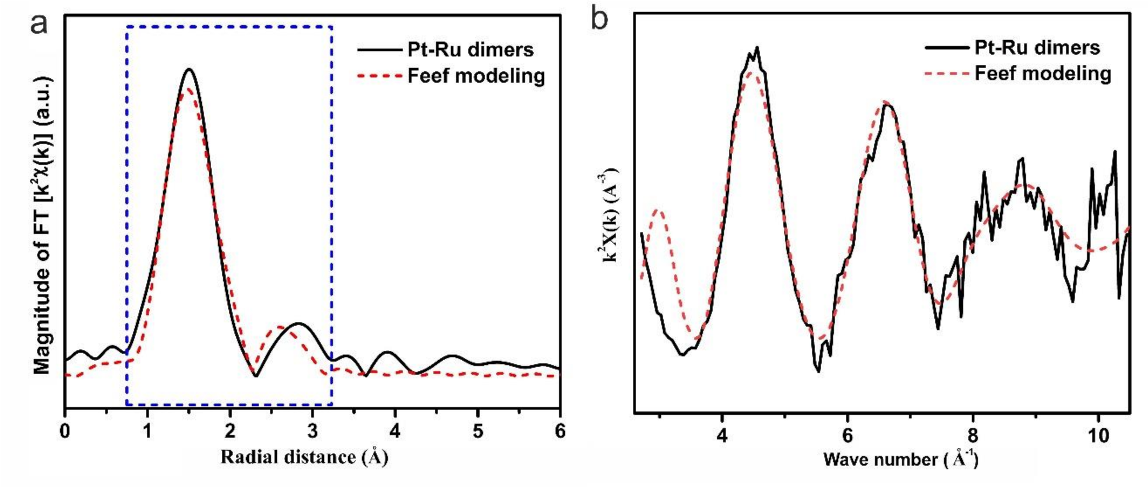

Supplementary Figure 28. Ru R space and K space curve fitting on Pt-Ru dimers. (a) Comparison in the magnitude of FT between Ru K edge experimental data from Pt-Ru dimer and the Feff R space fit; and (b) Comparison between experimental data and the Feff modeling based on the R space curve fitting result.

Supplementary Table 1. Comparison of HER performance of the Pt-Ru dimer catalysts with state-of-art Pt catalysts.

Catalyst	Overpotential /mV	Mass activity / A mg^{-1}	Supplementary References
Pt-Ru dimer	50	23.1	Our work
ALD50Pt/NGNs	50	10.1	1
Pt@DNA−GC	45	1.33	2
Pt_3Ni_2 NWs-S/C	70	3.3	3
PyPOP-Pt@G	100	1.3	4
Pt_1/MC	65	10	5
PtSA-NT-NF	50	0.07	6
Pt-GDY2	50	~10	7
PtNi-O/C	70	7.23	8
Pt SASs/AG	50	22.4	9

Supplementary Table 2. Adsorption energy of 1H on Ru atom of Pt(nH)Ru(mH).

Ru	Pt(0H)	Pt(1H)	Pt(2H)	Pt(3H)
Ru(0H)	**0**	-2.5	—	—
Ru(1H)	**-3.3**	**-1.2**	**-1.16**	**-0.85**
Ru(2H)	—	-0.53	-0.80	**-0.91**
Ru(3H)	—	-0.94	-0.64	**-0.80**

Supplementary Table 3. Gibbs free energy of H on Ru atoms of Pt(nH)Ru(mH).

Ru	Pt(1H)	Pt(2H)	Pt(3H)
Ru(2H)	-0.82	-0.44	0.85
Ru(3H)	1.68	0.08	**0.01**

.

Supplementary Table 4. The occupation number of d-orbital electrons of Pt and Ru of Pt(2H)Ru(3H) and Pt(3H)Ru(3H). The bold number represent the occupied d-orbitals.

	dxy(up)	dxy(down)	dyz(up)	dyz(down)	dz2(up)	dz2(down)	dxz(up)	dxz(down)	Dx2-y2(up)	Dx2-y2(down)
32-Ru	**0.92**	0.45	0.75	0.60	**0.92**	0.53	**0.90**	0.49	**0.91**	0.42
23-Ru	**0.86**	0.5	0.71	0.65	**0.91**	0.51	0.72	0.61	**0.86**	0.56
3-Ru	**0.91**	0.39	0.68	0.65	**0.90**	0.57	0.79	0.55	**0.88**	0.58

Supplementary References

1. Cheng, N. et al. Platinum single-atom and cluster catalysis of the hydrogen evolution reaction. *Nat. Commun.* **7**, 13638 (2016)..

2. Anantharaj, S., Karthik, P. E., Subramanian, B. & Kundu, S. Pt Nanoparticle anchored molecular self-assemblies of DNA: an extremely stable and efficient HER electrocatalyst with ultralow Pt content. *ACS Catal.* **6**, 4660−4672 (2016).

3. Wang, P. et al. Precise tuning in platinum-nickel/nickel sulfide interface nanowires for synergistic hydrogen evolution catalysis. *Nature Commun.* **8**, 14580 (2017).

4. Soliman, A. B. et al. Pt immobilization within a tailored porous-organic polymer–graphene composite: opportunities in the hydrogen evolving reaction. *ACS Catal.* **7**, 7847−7854 (2017).

5. Wei, H. et al. Iced photochemical reduction to synthesize atomically dispersed metals by suppressing nanocrystal growth. *Nature Commun.* **8**, 1490 (2017).

6. Zhang, L., Han, L., Liu H., Liu, X. & Luo, J. Potential-cycling synthesis of single platinum atoms for efficient hydrogen evolution in neutral media. *Angew. Chem. Int. Ed.* **56**, 13694 –13698 (2017).

7. Yin, X.-P. et al. Engineering the coordination environment of single-atom platinum anchored on graphdiyne for optimizing electrocatalytic hydrogen evolution. *Angew. Chem. Int. Ed.* **57**, 9382 –9386 (2018).

8. Zhao, Z. et al. Surface-engineered PtNi-O nanostructure with record-high performance for electrocatalytic hydrogen evolution reaction *J. Am. Chem. Soc.* **140**, 9046−9050 (2018).

9. Ye. S. et al. Highly stable single Pt atomic sites anchored on aniline-stacked graphene for hydrogen evolution reaction. *Energy Environ. Sci.* **12**, 1000−1007 (2019).
